# Stratified glucose-lowering response to vildagliptin and pioglitazone by obesity and hypertriglyceridemia in a randomized crossover trial

**DOI:** 10.3389/fendo.2022.1091421

**Published:** 2023-01-09

**Authors:** Rebecca Brandon, Yannan Jiang, Rui Qian Yeu, Ry Tweedie-Cullen, Kate Smallman, Glenn Doherty, Kerry A. Macaskill-Smith, Rebekah J. Doran, Penny Clark, Allan Moffitt, Troy Merry, Norma Nehren, Frances King, Jennie Harré Hindmarsh, Megan Patricia Leask, Tony R. Merriman, Brandon Orr-Walker, Peter R. Shepherd, Ryan Paul, Rinki Murphy

**Affiliations:** ^1^ Department of Medicine, Faculty of Medical and Health Sciences, The University of Auckland, Auckland, New Zealand; ^2^ Maurice Wilkins Centre for Molecular Biodiscovery, Auckland, New Zealand; ^3^ Department of Statistics, Faculty of Sciences, The University of Auckland, Auckland, New Zealand; ^4^ National Institute for Health Innovation, School of Population Health, The University of Auckland, Auckland, New Zealand; ^5^ Diabetes Foundation Aotearoa, Auckland, New Zealand; ^6^ Tongan Health Society, Auckland, New Zealand; ^7^ Ventures/Pinnacle Incorporated, Hamilton, New Zealand; ^8^ Procare Primary Health Organisation, Auckland, New Zealand; ^9^ Discipline of Nutrition, University of Auckland, Auckland, New Zealand; ^10^ Te Hiku Hauora, Northland District Health Board, Kaitaia, New Zealand; ^11^ Ngāti Porou Hauora, Tairāwhiti, New Zealand; ^12^ Department of Biochemistry, University of Otago, Dunedin, New Zealand; ^13^ Division of Clinical Immunology and Rheumatology, University of Alabama at Birmingham, Birmingham, AL, United States; ^14^ Middlemore Clinical Trials, Auckland, New Zealand; ^15^ Department of Molecular Medicine and Pathology, School of Medical Sciences, The University of Auckland, Auckland, New Zealand; ^16^ Department of Medicine, University of Waikato, Waikato, New Zealand

**Keywords:** dipeptidyl peptidase inhibitor, Māori, Pacific, pioglitazone, precision medicine, stratified drug response, thiazolidinedione, obesity

## Abstract

**Background:**

Understanding which group of patients with type 2 diabetes will have the most glucose lowering response to certain medications (which target different aspects of glucose metabolism) is the first step in precision medicine.

**Aims:**

We hypothesized that people with type 2 diabetes who generally have high insulin resistance, such as people of Māori/Pacific ethnicity, and those with obesity and/or hypertriglyceridemia (OHTG), would have greater glucose-lowering by pioglitazone (an insulin sensitizer) versus vildagliptin (an insulin secretagogue).

**Methods:**

A randomised, open-label, two-period crossover trial was conducted in New Zealand. Adults with type 2 diabetes, HbA1c>58mmol/mol (>7.5%), received 16 weeks of either pioglitazone (30mg) or vildagliptin (50mg) daily, then switched to the other medication over for another 16 weeks of treatment. Differences in HbA1c were tested for interaction with ethnicity or OHTG, controlling for baseline HbA1c using linear mixed models. Secondary outcomes included weight, blood pressure, side-effects and diabetes treatment satisfaction.

**Results:**

346 participants were randomised (55% Māori/Pacific) between February 2019 to March 2020. HbA1c after pioglitazone was lower than after vildagliptin (mean difference -4.9mmol/mol [0.5%]; 95% CI -6.3, -3.5; p<0.0001). Primary intention-to-treat analysis showed no significant interaction effect by Māori/Pacific vs other ethnicity (1.5mmol/mol [0.1%], 95% CI -0.8, 3.7), and per-protocol analysis (-1.2mmol/mol [0.1%], 95% CI -4.1, 1.7). An interaction effect (-4.7mmol/mol [0.5%], 95% CI -8.1, -1.4) was found by OHTG status. Both treatments generated similar treatment satisfaction scores, although there was greater weight gain and greater improvement in lipids and liver enzymes after pioglitazone than vildagliptin.

**Conclusions:**

Comparative glucose-lowering by pioglitazone and vildagliptin is not different between Māori/Pacific people compared with other New Zealand ethnic groups. Presence of OHTG predicts greater glucose lowering by pioglitazone than vildagliptin.

**Clinical trial registration:**

www.anzctr.org.au, identifier (ACTRN12618001907235).

## Introduction

The epidemic of type 2 diabetes (T2D) is of significant concern due to increased morbidity, mortality, and health care costs. Current treatment guidelines recommend early use of sodium glucose transport protein 2 inhibitors (SGLT2i) and glucagon like peptide 1 receptor agonists (GLP1RA) for those with high cardiovascular risk, heart failure or renal disease (1). In the remainder, metformin, followed by a range of other medications are recommended for glucose lowering on a sequential, additive basis, influenced by cost, side effect profiles, and average treatment effects from clinical trials (1). Consideration of personal characteristics such as comorbidities, and circumstances such as food security, financial stability, alongside setting an appropriate target HbA1c for individuals is part of individualising treatment of T2D. Identifying subgroups or strata of patients with similar characteristics who respond better to one type of glucose-lowering therapy over another, and/or have altered risk of treatment-specific side effects, has been termed stratification of treatment response, which represents the first step towards precision medicine for T2D (2).

The glucose lowering response to non-insulin therapies that target discrete aspects of glucose metabolism is, extremely variable between individuals with T2D. Knowledge of which medications are likely to be most effective for individuals with T2D is important to minimise cost, side effects and periods of hyperglycaemia associated with initiating an ineffective medication. In the absence of knowledge about which subgroups of people with T2D respond better to a particular medication, relatively low glucose lowering response to certain treatments may be inappropriately attributed to non-adherence. There is emerging evidence for stratification of glucose lowering response to certain medications for T2D by baseline characteristics, such as ethnicity, obesity and triglyceride levels.

Ethnicity as a marker for differential medication responsiveness has been investigated as a simple tool for therapeutic stratification of T2D medications. Both SGLT2i and dipeptidyl peptidase 4 inhibitor (DPP4i) have been reported to be more effective in lowering HbA1c in people of Asian compared with European ethnicity (3). Metformin may be more effective in African Americans with younger onset T2D compared to Americans of European ethnicity (4). Alpha-glucosidase inhibitors appear to work better in South East Asians than Europeans (5).

It is unclear whether differences in treatment response by ethnicity are due to biological differences in underlying T2D aetiology, or whether ethnicity serves as a proxy for residual confounders such as level of glycaemia, concomitant diabetes therapies, dietary factors, medication adherence or other health inequities. Biological differences in underlying T2D aetiology using routinely assessed clinical markers of insulin resistance, such as obesity and/or high triglycerides (OHTG), have been shown to predict less glucose-lowering to DPP4i (6), which amplifies glucose-stimulated beta cell insulin secretion through the endogenous incretin pathway. Those with obesity have been reported to have greater glucose-lowering with thiazolidinediones (6, 7), which act to improve insulin sensitivity and lipid metabolism by stimulating the nuclear receptor peroxisome proliferator-activated receptor gamma (PPARG).

Māori are the indigenous people of Aotearoa, New Zealand and share common Polynesian ancestry with Pacific peoples. Obesity and elevated triglyceride levels are prevalent in people of Māori and Pacific ethnicities (8, 9). There are disparities in diabetes outcomes for Māori and Pacific peoples (10), hence research into biological and other factors underlying these disparities are needed. To date glucose lowering response to different medications in people of Māori or Pacific ethnicity with T2D has not been studied. In this T2D medication, Which One is Right Here (WORTH) trial, we tested the potential stratification of comparative glycaemic response to pioglitazone (thiazolidinedione) and vildagliptin (DPP4i), by Māori or Pacific ethnicity and by OHTG status.

## Materials and methods

### Participant eligibility

Patients with T2D for >1 year, who had been on stable doses of metformin and/or sulfonylurea for >3 months were eligible to participate if they were aged 18–80 years inclusive, had HbA1c > 58 mmol/mol [> 7.5%] and < 111 mmol/mol [<12.3%], had never been on DPP4i or thiazolidinedione, had no insulin use, had no active infection requiring antibiotics, had no contraindication to either trial medication, and were able to give written informed consent. These eligibility criteria were modified to enable those on stable doses of two other types of oral glucose-lowering medications to participate in this trial (acarbose or dapagliflozin) as dual therapy with either metformin or sulfonylurea. Recruitment targeted 40% of participants being of Māori and/or Pacific ethnicity.

### Settings and location

The trial participants were recruited at nine sites across urban (Auckland and Waikato) and rural settings (Te Tai Tokerau (Northland) and Te Tairāwhiti (East Coast of the North Island) in NZ.

### Trial design

We conducted an investigator-initiated, multi-centre, open-label, two-treatment (pioglitazone 30mg once daily [P], vildagliptin 50mg once daily [V]), two-period (16-weeks each), randomised, crossover trial. There was no washout period between medications because medication withdrawal could have caused detrimental rebound hyperglycaemia and this was deemed unnecessary due to neither of the two medications having a continuing glucose lowering effect beyond 4 weeks. The primary outcome of HbA1c measurement, which reflects glycaemia over the preceding 8-12 weeks, would therefore reflect results only from the last medication during the 16-week treatment period, without any carry over effect from the first medication. The strength of the PV/VP crossover design is that both interventions were evaluated for each participant, which allows comparison at the individual rather than group level alone (11). In addition, participants could express preferences by comparing their experiences of both interventions which is not possible in a parallel group design. The trial protocol was approved by the NZ Health and Disability Ethics Committee, and has been published (12). All participants gave written informed consent.

### Outcome and baseline measures

The primary outcome measure was HbA1c after each 16-week treatment period. The primary analysis was to test stratification in HbA1c response by Māori or Pacific ethnicity and the pre-specific secondary analysis was to test stratification in HbA1c response by OHTG status. To minimise confounding by adherence or treatment change, protocol adherence was considered to be those who on assessment had >75% adherence to the trial medication based on pill counts, had not commenced any additional glucose-lowering therapies since the baseline eligibility visit, and had not stopped any baseline glucose-lowering medication. HbA1c data were considered invalid if they were obtained more than 7 days after completing the study medication phase. Secondary outcome measures included body weight, blood pressure (BP), frequency of side effects, and Diabetes Treatment Satisfaction Questionnaire (DTSQ) (13) total scores, change in scores, and patient preference for either medication at the end of each treatment period. Serious adverse events (AEs) were collected throughout the trial period.

Self-reported ethnicity was recorded at the baseline visit. Participants were asked to tick all of the following categories that applied: Māori, Pacific, NZ European, Other European, Indian, Other Asian, Other (asked to specify). Prioritised ethnicity classification as Māori or Pacific was defined if either of these ethnicities were ticked or specified in “Other”. Diabetes diagnosis date, current and past diabetes medications, co-morbidities and other medications were verified by medical records. Fasting blood tests were performed to assess HbA1c, glucose, lipids, renal, liver function at baseline and were repeated at the end of each treatment period. OHTG was defined by the presence of BMI ≥30kg/m^2^ and/or high triglycerides ≥2.3mmol/L. Fasting C-peptide, diabetes autoantibodies and blood for genetic analysis were taken at baseline. All biochemical analyses were performed at local diagnostic laboratories with validated and accredited assays. The 3-screen islet cell autoantibody Enzyme-Linked Immunosorbent Assay (ELISA) kit (RSR limited, Cardiff, UK) was used for combined quantitative determination of glutamic acid decarboxylase (GAD), islet tyrosine phosphatase-2 (IA-2) and zinc transporter8 (ZnT8) autoantibodies in human serum. All samples testing above the assay concentration threshold of 20u/mL were tested again using separate ELISA assays for each autoantibody (Anti-GAD and Anti-IA2, Euroimmun AG, Lübeck, Germany; ZnT8, ElisaRSR, Cardiff, UK).

### Randomisation

Eligible participants were randomised 1:1 to one of the two sequences (VP or PV) by a central trial pharmacist using a secure trial database. The randomised study medication was couriered to the participant’s nominated address by the central trial pharmacy. Randomisation lists were prepared by the trial statistician, using permuted block randomisation with variable block sizes (2 or 4) and stratified by recruiting region and self-reported ethnicity (Māori and Pacific vs non-Māori/non-Pacific, including New Zealand European, other European, Indian, Asian, and other groups). This was an open-label trial, where both participants and research staff were aware of the treatment sequences [VP/PV] after randomisation.

### Statistical analysis

We aimed to recruit a total of 300 participants, with a target sample size of 40% Māori and Pacific people. This sample size would provide 80% power at 5% significance level to detect a minimal effect size of 0.35 standard deviation (SD) between Māori and Pacific vs non-Māori/non-Pacific groups on the difference in HbA1c between two test medications, allowing for 10% loss to follow up. Full details were reported in the published trial protocol (12).

All randomised participants were included in the primary outcome analysis, following the intention to treat (ITT) principle. Missing HbA1c data after each treatment were imputed using the baseline value or any lab data collected post-baseline before the scheduled assessment window. Per protocol (PP), analysis was also performed on the primary outcome, including only those participants who provided complete outcome data with no major protocol violations. All outcomes were assessed using valid visit data collected within the scheduled assessment window. No imputation was considered on secondary outcomes. Statistical analyses were performed using SAS version 9.4 (SAS Institute Inc., Cary, NC, USA).

Baseline demographic and clinical characteristics of all randomised participants were summarised overall and by ethnicity. Continuous variables were presented as mean and SD. Categorical variables were presented in frequencies and percentages. Whether the difference in achieved HbA1c for the two medications was different between Māori and Pacific vs non-Māori/non-Pacific participants was tested using a linear mixed model with both fixed and random effects. The fixed effects included the baseline outcome value, period, medication class, patient group and its interaction with the medication. The random effect included patients as the cluster. The model-adjusted mean difference between two medications was estimated for each patient group, and the interaction effect between medications and patient groups was estimated with a 95% confidence interval (CI). Similar regression analyses were conducted to test the medication response with other patient groups of interest, including OHTG status (high BMI ≥30kg/m^2^ and/or high triglycerides ≥2.3mmol/L vs BMI <30kg/m^2^ and triglycerides <2.3mmol/L). As a secondary analysis, the overall treatment effect was compared between the two medications without the interaction with patient group term and potential carryover effect was tested. Generalised linear regression was used on other secondary outcomes collected at the end of the trial, including patient medication preference and DTSQ change score. Statistical tests were two-sided at 5% significance level. The CONSORT 2010 statement extension for randomised crossover trials was followed (14).

## Results

### Participant flow

**Figure 1:**
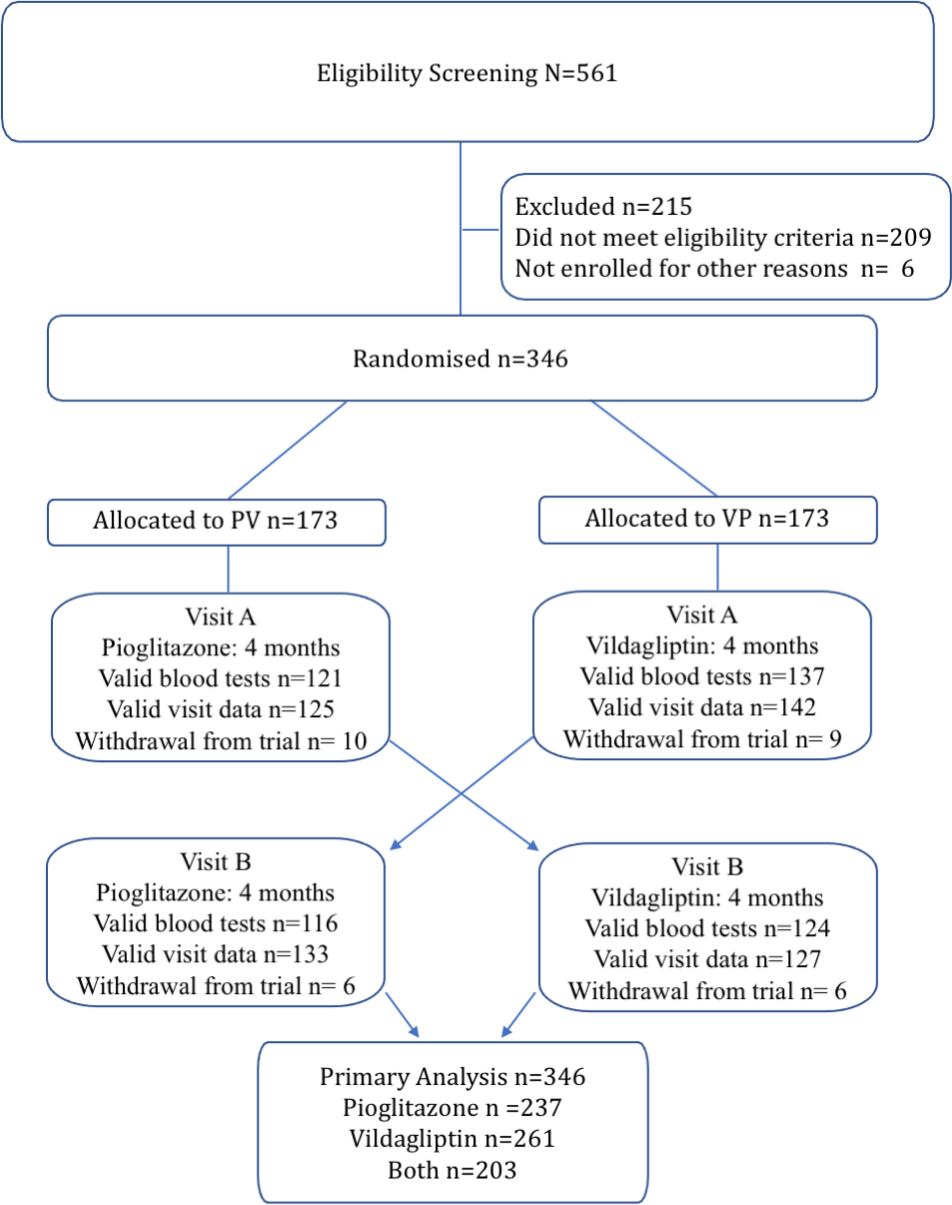
Flow diagram of the study participants included in this analysis.

A total of 346 participants who completed eligibility screening between February 2019 and March 2020, were randomised to VP or PV sequence (173 each). Of these, valid HbA1c data were obtained in 261 participants after vildagliptin and 237 participants after pioglitazone, with 203 participants completing both assessments (**Figure 1**). The large proportion of invalid HbA1c data was due to pandemic related delays in obtaining timely blood tests within 7 days of completing the study medication phase. There were 16 participants who withdrew during pioglitazone treatment and 15 who withdrew during vildagliptin treatment.

### Baseline data

The mean (SD) for age was 57.5 (10.9) years, HbA1c 75 (12) mmol/mol), [9% 1.1)], diabetes duration 9 (6) years; 41% of participants were women, 55% were Māori and/or Pacific (**Table 1**). Of all trial participants, 78 (23%) were Māori, 111 (32%) were Pacific people, 90 (26%) were NZ European, 67 (26%) were from other ethnic groups. There was no difference in characteristics by sequence PV vs VP. OHTG was present in 88% of Māori and Pacific participants. (**Table 2**). Nine participants tested positive in the triple autoantibody screening assay, verified as only GAD, (**Supplementary Table 1**). All had preserved fasting C-peptide (320-1983pmol/L), and were included in the analyses.

**Table 1:** Baseline demographics and clinical characteristics.

	Overall	Māori and Pacific	Non Māori and non-Pacific
	n=346	n=189 (55%)	n=157 (45%)
Age (years)	57.5 (10.9)	56.2 (10.9)	59.1 (10.7)
Sex			
Female	141 (40.8%)	92 (48.7%)	49 (31.2%)
Male	205 (59.2%)	97 (51.3%)	108 (68.8%)
OHTG			
Yes	273 (78.9%)	166 (87.8%)	107 (68.2%)
No	72 (20.8%)	22 (11.7%)	50 (31.8%)
Missing	1 (0.3%)	1 (0.5%)	0 (0%)
Duration of diabetes (years)	9.0 (6.3)	8.4 (6.4)	9.6 (6.1)
Current smoker	49 (14.2%)	36 (19.0%)	13 (8.3%)
Baseline diabetes medication			
Metformin	339 (98.0%)	186 (98.4%)	153 (97.5%)
Sulfonylureas	216 (62.4%)	120 (63.5%)	96 (61.1%)
Other*	13 (3.8%)	7 (3.7%)	6 (3.8%)
BMI (kg/m^2^)	35.5 (7.8)	38.0 (7.9)	32.5 (6.6)
Mean BP systolic (mmHg)	132.6 (15.1)	131.4 (15.6)	133.99 (14.4)
Mean BP diastolic (mmHg)	80.9 (8.5)	80.7 (8.8)	81.20 (8.3)
Fasting Glucose (mmol/L)	10.5 (3.0)	10.5 (3.1)	10.46 (2.9)
Fasting TG (mmol/L)	2.1 (1.5)	2.1 (1.7)	2.00 (1.3)
Creatinine (umol/L)	81.1 (18.5)	81.6 (20.1)	80.5 (16.3)
Fasting C-peptide (pmol/L)	1162 (494)	1201 (469)	1117 (520)
GAD antibodies	n=7/341 (2.1%)	n=4/185 (2.2%)	n=3/156 (1.9%)

Data are n (%) or mean (SD). OHTG refers to obese (BMI ≥ 30kg/m^2^) and/or high triglycerides > 2.3mmol/L (TG). GAD, Glutamic acid decarboxylase.

*Other medication includes acarbose in 10 cases and dapagliflozin in 3 cases.

**Table 2:** Descriptive data at baseline and after each treatment.

	Baseline (B)	Pioglitazone (P)	Vildagliptin (V)
	N, mean (SD)	N, mean (SD)	N, mean (SD)
HbA1c in mmol/mol [%]			
Overall cohort	346, 74·9 (11·5) [9.0]	237, 60·6 (12·1) [7.7]	261, 65·8 (13·4) [8.2]
Māori/Pacific	189, 76·0 (12·2) [9.1]	114, 61·6 (12·5) [7.8]	135, 67·4 (15·0) [8.3]
Non Māori/Pacific	157, 73·5 (10·6) [8.9]	123, 59·6 (11·6) [7.6]	126, 64·1 (11·3) [8.0]
OHTG	273, 75·5 (11·4) [9.1]	184, 60·7 (12·6) [7.7]	206, 67·0 (13·9) [8.3]
No OHTG	72, 72·6 (11·9) [8.8]	52, 60.2 (10.3) [7.7]	54, 61.4, (10.3) [7.8]
Female	141, 75·6 (11·9) [9.1]	97, 58.5 (12·1) [7.5]	109, 65.4 (13.2) [8.1]
Male	205, 74.3 (11.3) [8.9]	140, 62.0 (11.9) [7.8]	152, 66.2 (13.5) [8.2]
Weight in kilograms			
Overall cohort	346, 103.0 (24.8)	222, 105.5 (26.9)	235, 103.2 (26.1)
Māori/Pacific	189, 109.3 (25.4)	107, 113.7 (29.4)	116, 112.1 (28.5)
Non Māori/Pacific	157, 95.3 (21.8)	115, 97.8 (21.9)	119, 94.6 (20.1)
OHTG	273, 109.7 (23.2)	173, 113.1 (25.3)	181, 110.8 (24.6)
No OHTG	72, 77.6 (9.6)	49, 78.6 (10.6)	53, 77.6 (9.7)
Female	141, 99.5 (23,5)	92, 102.6 (26.2)	92, 99.5 (24.5)
Male	205, 105.3 (25.4)	130, 107.5 (27.4)	143, 105.7 (26.9)
Systolic blood pressure in mmHg			
Overall cohort	346, 132.6 (15.1)	220, 129.6 (15.3)	231, 130.9 (13.9)
Māori/Pacific	189, 131.4 (15.6)	107, 128.8 (16.8)	114, 129.6 (14.4)
Non Māori/Pacific	157, 134.0 (14.4)	113, 130.4 (13.8)	117, 132.1 (13.4)
OHTG	273, 133.0 (14.8)	172, 130.1 (14.4)	179, 130.6 (13.2)
No OHTG	72, 131.2 (16.1)	48, 128.0 (18.4)	51, 132.0 (16.3)
Female	141, 131.2 (15.3)	90, 128.9 (14.8)	89, 129.3 (13.1)
Male	205, 133.5 (14.9)	130, 130.1 (15.8)	142, 131.9 (14.4)
Diabetes Treatment Satisfaction Questionnaire (DTSQ) total score*			
Overall cohort	346, 28.6 (6.0)	256, 30.5 (5.9)	268, 30.0 (6.5)
Māori/Pacific	189, 29.4 (6.0)	128, 30.3 (6.0)	133, 30.1 (6.8)
Non Māori/Pacific	157, 27.6 (5.8)	128, 30.7 (5.8)	135, 30.0 (6.3)
OHTG	273, 28.2 (6.2)	198, 30.2 (6.2)	208, 29.8 (6.6)
No OHTG	72, 29.8 (5.0)	57, 31.4 (4.5)	59, 30.8 (6.2)
Female	141, 28.9 (6.4)	111, 31.1 (5.7)	110, 30.4 (6.9)
Male	205, 28.3 (5.6)	145, 30.0 (6.0)	158, 29.8 (6.3)

*Questions contained in the DTSQ are shown in **Table 5**.

### Outcomes

Overall, a greater mean decrease in HbA1c was observed after pioglitazone than after vildagliptin treatment (adjusted mean difference -4.9mmol/mol [-0.5%], 95%CI -6.3, -3.5; p < 0.0001) (**Table 3**). No carry-over effect was found (p=0.94). The primary ITT analysis showed no interaction effect in HbA1c response between treatments (pioglitazone vs vildagliptin) by ethnicity (Māori and Pacific -2.1mmol/mol [0.19%] vs non-Māori/non-Pacific -3.6 mmol/mol [0.33%]; interaction effect 1.5mmol/mol [0.14%], 95%CI -0.8, 3.7, p= 0.2). This was consistent with the PP analysis (Māori and Pacific -6.1mmol/mol [0.56%] vs non-Māori/non-Pacific -4.9mmol/mol [0.45%]; interaction effect -1.2mmol/mol [0.11%], 95%CI -4.1, 1.7, p=0.40); and the analysis of all valid data (Māori and Pacific -5.4mmol/mol [0.5%] vs non-Māori/non-Pacific -4.4mmol/mol [0.4%], interaction effect -1.1mmol/mol [0.1%], 95%CI -3.8, 1.7; p=0.4) (**Table 4**).

**Table 3:** The estimated medication effects on HbA1c and its interaction with ethnicity (primary outcome analysis).

HbA1c	Pioglitazone versus Vildagliptin (PV)		Difference in PV between Ethnicity	
	Mean estimate (95% CI)	p-value	Mean estimate (95% CI)	p-value
ITT analysis				
Māori/Pacific (mmol/mol)	-2.1 (-3.6,-0.6)	0.0070	1.5 (-0.8, 3.7)	0.2
non-Māori/Pacific (mmol/mol)	-3.6 (-5.2, -1.9)	< 0.0001		
Māori/Pacific (%)	-0.19 (-0.33, -0.05)	0.0070	0.14 (-0.07, 0.34)	0.2
non-Māori/Pacific (%)	-0.33 (-0.48, -0.17)	< 0.0001		
PP analysis				
Māori/Pacific (mmol/mol)	-6.1 (-8.2,-4.0)	<0.0001	-1.2 (-4.1, 1.7)	0.4
non-Māori/Pacific (mmol/mol)	-4.9 (-6.9, -2.9)	<0.0001		
Māori/Pacific (%)	-0.56 (-0.75, -0.37)	<0.0001	0.01 (0, 0.03)	0.4
non-Māori/Pacific (%)	-0.45 (-0.63, -0.27)	<0.0001		
**HbA1c**	**Pioglitazone**	**Vildagliptin**	**Pioglitazone versus Vildagliptin (PV)**	
	**Mean estimate (95% CI)**	**Mean estimate (95% CI)**	**Mean estimate (95% CI)**	**p-value**
Overall medication effect (mmol/mol)	61.2 (59.8, 62.5)	66.1 (64.7, 67.4)	-4.9 (-6.3, -3.5)	<0.0001
Overall medication effect (%)	7.8 (7.6, 7.9)	8.2 (8.1, 8.3)	-0.45 (-0.58, -0.32)	<0.0001

ITT, intention to treat; PP, per protocol; CI, confidence interval.

**Table 4:** The estimated medication effects on patient outcomes and its interaction with patient groups.

	Pioglitazone versus Vildagliptin (PV)		Difference in PV between Patient groups	
	Mean estimate (95% CI)	p-value	Mean estimate (95% CI)	p-value
HbA1c (mmol/mol)				
Māori/Pacific	-5.4 (-7.4, -3.5)	<0.0001	-1.1 (-3.8, 1.7)	0.4
non-Māori/Pacific	-4.4 (-6.3, -2.5)	<0.0001		
OHTG	-5.9 (-7.5, -4.4)	<0.0001	-4.7 (-8.1, -1.4)	0.005
non-OHTG	-1.2 (-4.1, 1.8)	0.4		
HbA1c (%)				
Māori/Pacific	-0.49 (-0.68, -0.32)	<0.0001	-0.10 (-0.35, 0.16)	0.4
non-Māori/Pacific	-0.40 (-0.58, -0.23)	<0.0001		
OHTG	-0.54 (-0.69, -0.40)	<0.0001	-0.43 (-0.74, -0.13)	0.005
non-OHTG	-0.11 (-0.38, 0.16)	0.4		
Weight (kg)				
Māori/Pacific	1.6 (0.9, 2.3)	<0.0001	0.1(-0.9, 1.0)	0.9
non-Māori/Pacific	1.5 (0.9, 2.2)	<0.0001		
OHTG	1.6 (1.1, 2.2)	<0.0001	0.2 (-0.9, 1.4)	0.7
non-OHTG	1.4 (0.4, 2.4)	0.007		
Systolic blood pressure (mmHg)				
Māori/Pacific	0.1 (-2.9, 3.2)	0.9	0.9 (-3.3, 5.1)	0.7
non-Māori/Pacific	-0.8 (-3.7, 2.1)	0.6		
OHTG	0.1 (-2.2, 2.5)	0.9	2.4 (-2.7, 7.4)	0.4
non-OHTG	-2.2 (-6.7, 2.3)	0.3		
Diabetes Treatment Satisfaction Questionnaire (DTSQ) total score*				
Māori/Pacific	0.2 (-1.0, 1.5)	0.7	-0.5 (-2.2, 1.2)	0.6
non-Māori/Pacific	0.7 (-0.5, 1.9)	0.2		
OHTG	0.4 (-0.6, 1.4)	0.4	-0.1 (-2.2, 2.0)	0.9
non-OHTG	0.5 (-1.3, 2.4)	0.6		

All valid patient data collected at baseline and after each medication treatment were used in the analysis; missing data were not imputed.

*Questions contained in the DTSQ are shown in **Table 5**.

A treatment difference in HbA1c response was observed in participants with OHTG (-5.9mmol/mol [0.54%]) compared with those without OHTG (-1.2mmol/mol [0.11%]), with an estimated interaction effect of -4.7mmol/mol [0.43%], 95%CI -8.1, -1.4, p=0.005 (**Table 4**).

Mean weight after pioglitazone was higher than after vildagliptin treatment, with an adjusted mean difference of 1.6kg (95%CI [1.1, 2.0]; p<0.0001). There was no interaction in weight change response to pioglitazone versus vildagliptin by ethnicity or OHTG status (**Table 4**). BP and DTSQ did not differ by treatment group in the overall analysis, by ethnicity, or OHTG status (**Tables 4**, **5**). There was no significant carryover effect detected for weight (p=0.66). There were 27 (7.8%) participants reporting new or worsening pedal edema after pioglitazone and 15 (4.3%) after vildagliptin (Chi-square p=0.06).

**Table 5:** DTSQ scores at baseline and after each treatment.

	DTSQ scores as mean (SD)		
	Baseline (B)	Pioglitazone (P)	Vildagliptin (V)
Overall DTSQ score*	28.6 (6.0) N=345	30.6 (5.8) N=232	29.9 (6.6) N=245
1.How satisfied are you with your current treatment	4.7 (1.4) N=346	5.0 (1.4) N=232	4.9 (1.4) N=245
2. How often have you felt your blood sugars have been unacceptably high recently?	3.3 (2.0) N=346	1.3 (1.7) N=243	1.7 (1.9) N=256
3. How often have you felt your blood sugars have been unacceptably low recently?	1.1 (1.6) N=346	0.7 (1.2) N=243	0.6 (1.2) N=256
4. How convenient have you been finding your treatment recently?	4.9 (1.4) N=346	5.3 (1.2) N=243	5.1 (1.5) N=256
5. How flexible have you been finding your treatment recently?	4.6 (1.5) N=346	5.2 (1.3) N=243	5.1 (1.4) N=256
6. How satisfied are you with your understanding of your diabetes?	4.9 (1.3) N=346	5.1 (1.3) N=243	5.2 (1.1) N=256
7. Would you recommend this form of treatment to someone else with your kind of diabetes?	4.8 (1.7) N=345	4.9 (1.8) N=243	4.7 (1.9) N=256
8. How satisfied would you be to continue with your present form of treatment?	4.6 (1.6) N=346	5.0 (1.6) N=232	4.9 (1.7) N=245

*Derived from the sum of responses to question 1 and questions 4-8 each rated on a scale from 0 to 6, with a high score (maximum 36) representing high treatment satisfaction.

Hepatic enzymes, alanine aminotransferase (ALT), aspartate aminotransferase (AST), gamma-glutamyl transferase (GGT) were lower after pioglitazone but remained unchanged after vildagliptin (difference p<0.0001). The difference in hepatic enzymes after pioglitazone vs vildagliptin, did not differ significantly by Māori and Pacific ethnicity or OHTG. Triglycerides reduced after pioglitazone compared with after vildagliptin (mean difference -0.3mmol/L, p<0.0001), with no interaction by ethnicity or OHTG grouping. HDL-C increased more after pioglitazone than after vildagliptin (+0.1mmol/L, p<0.0001).

At the final trial visit, 257 participants indicated their preferred medication: 98 (38%) preferred pioglitazone, 87 (34%) preferred vildagliptin, and 72 (28%) indicated either no preference (n=62) or neither (n=10). No differences in medication preference were found by ethnicity, or OHTG strata after adjusting for their treatment sequence (PV or VP), the change in HbA1c, weight and DTSQ score between two treatments.

There were 15 serious adverse events: 3 deaths (2 strokes and 1 myocardial infarction) and 12 hospitalisations. None were deemed to be due to the trial medication by an independent data safety monitoring committee.

## Discussion

This multicentre, two-way, randomised crossover trial showed that the glucose-lowering response to pioglitazone relative to vildagliptin was not different between people of Māori or Pacific ethnicity and other New Zealand ethnicities. However, there was a significant interaction in relative glucose-lowering by OHTG status, with 4.7mmol/mol greater reduction in HbA1c by pioglitazone relative to vildagliptin among those with OHTG. Those without OHTG had similar glucose lowering to each medication. Importantly, both treatments generated similar treatment satisfaction scores although there was greater weight gain and greater improvement in lipids and liver enzymes after pioglitazone than vildagliptin. No severe adverse events were directly related to these treatments.

While people of Māori or Pacific ethnicity had higher prevalence of OHTG, such ethnicity grouping did not predict altered glucose lowering response to pioglitazone versus vildagliptin. Within Māori and Pacific populations, there is genetic heterogeneity in risk and aetiology of T2D. A genetic variant (*rs373863828*, p.Arg457Gln) in the *CREBRF* gene, specifically found in approximately 25% people of Māori and Pacific ancestry, is associated with increased body mass index (15, 16) yet 40% lower odds of T2D (15, 16), due to enhanced insulin secretion capacity (17). Testing whether *CREBRF* gene variant status has utility in predicting stratified glucose lowering responses to T2D medications among people of Māori and Pacific ethnicity is currently underway (12). OHTG status has been reported to predict lower glucose response in studies with European participants (6).

In contrast to rosiglitazone, which is no longer used due to its associated increased risk of myocardial infarction and overall mortality (18), pioglitazone has demonstrable cardiovascular benefits (19, 20). This is thought to be partly due to its favourable impact on lipids (21, 22), which was also observed in this trial. In addition, our finding of improvement in liver enzymes with pioglitazone treatment is consistent with its reported benefits in non-alcoholic fatty liver disease and non-alcoholic steatohepatitis. Whilst the weight gain with pioglitazone has been highlighted as a caution for its use, our data suggests the weight gain was mild and did not reduce patient satisfaction.

The lack of interaction of reduction in liver enzymes or lipids after pioglitazone with baseline OHTG is interesting. One explanation is that the glucose lowering action of pioglitazone is mediated by ligand binding to nuclear receptor PPARG which are most abundant in adipocytes and hence greater in people with obesity (23). In contrast, the liver enzyme or lipid lowering action of pioglitazone may be mediated by other pathways that are not directly correlated with baseline OHTG status such as PPAR alpha expression (24).

Comparative medication glucose response data from this crossover study design provides evidence-based understanding of how patients would respond if they were to switch from one medication to another. While this is something that happens frequently in diabetes care, there is very limited clinical data about how patients’ glycaemic control changes when this happens or which medication they prefer. In addition, several consensus reports describe that when making treatment decisions for patients with type 2 diabetes it is important to consider not only the efficacy and safety of each medication, but also patient preference. This open-label cross-over study provides the added value of patient preference outcomes between pioglitazone and vildagliptin in the real-world setting. The protocol for a three-way crossover study including a DPP4 inhibitor, thiazolidinedione and a SGLT2 inhibitor has been described, which will provide further information on stratification of glucose response to three diabetes medications by routine clinical features ascertained at baseline (25).

A key limitation of this trial was the low follow up rate resulting in only 203 participants with valid HbA1c data after both treatments. Some of this related to difficulties encountered during the pandemic in obtaining a timely HbA1c result at the end of each study medication treatment phase and in conducting in person assessments. This is unlikely to have introduced any retention bias as each person contributed their own comparative data for both medications. Furthermore, the analysis was conducted with and without imputation on the primary analysis population, to test the robustness of the findings. Secondly, the open-label nature of the trial was selected for lower cost and complexity, which may have introduced observer and participant bias, but increased external validity. Thirdly, the crossover trial design might have led to the inappropriate estimation of the risk of weight gain with each medication, although the sensitivity analysis showed no significant carryover effect. The crossover design was selected as the best method to test differential glucose-lowering responses using HbA1c as the primary outcome measure between two diabetes medications in different patient subgroups (11). A longer treatment duration of each period could have mitigated against the slow onset and offset of each medication in absence of wash-out period. However, the 16-week consecutive treatment period was considered the most efficient study design.

It is well recognised that overall glucose lowering effects of pioglitazone are typically greater than vildagliptin. The important finding of this study is that a superior glucose-lowering response to pioglitazone versus vildagliptin is observed mainly in those with OHTG. Whilst OHTG is more prevalent in those of Māori and Pacific ethnicity, the glucose-lowering response was not significantly different by such ethnicity group in a multi-ethnic New Zealand population. This has several clinical implications. Firstly, these findings suggest that ethnicity is not a sufficient proxy for stratified medicine for pioglitazone and vildagliptin in T2D. Rather, clinical markers of insulin resistance that are routinely available in clinical practice such as BMI and triglyceride levels are able to predict whether superior glucose lowering response to pioglitazone relative to vildagliptin is expected. The recent progress in better defining novel T2D subtypes based on underpinning disease mechanisms such as age at diabetes diagnosis, GAD antibodies, C-peptide and genetic factors need to be explored for their utility in predicting other stratified glucose lowering responses (26). However, the use of non-routine biomarkers are less easily adopted in clinical practice. Secondly, the participant satisfaction data suggests people are generally not troubled by the small weight increase from pioglitazone so this medication is best selected for those with OHTG to maximise risk vs benefits. Finally, T2D treatment guidelines could include consideration of OHTG to inform treatment selection between vildagliptin and pioglitazone. Including a more prominent role for pioglitazone over vildagliptin in the presence of OHTG would be reasonable since this medication produces much greater glucose lowering in this subgroup. This is a robust finding that has now been replicated in a non-European population. Pioglitazone treatment selection will need to be considered in the context of other common indications for this medication such as fatty liver or dyslipidaemia, and relatively uncommon contraindications such as heart failure, osteoporosis and macular oedema, as part of individualised care based on patient characteristics.

## Data availability statement

The raw data supporting the conclusions of this article will be made available by the authors, without undue reservation.

## Ethics statement

The studies involving human participants were reviewed and approved by NZ Health and Disability Ethics Committee. The patients/participants provided their written informed consent to participate in this study.

## Author contributions

RM designed the trial. YJ was responsible for the statistical analysis of the data. TRM was responsible for the genetic analysis plan. ML designed the population-specific genetic variant panel. TM assisted with genetic analysis of the data. RM and PRS obtained funding. RB, RY, RT-C, KS, GD, KAM, RD, PC, AM, NN, FK, JH, BO-W, and RP were responsible for trial implementation and data acquisition. RB and RM wrote the first draft of this manuscript and RM handled the revisions. All authors reviewed the manuscript and approved the final version for submission.
