# Supplementary material for: Stratified glucose-lowering response to vildagliptin and pioglitazone by obesity and hypertriglyceridemia in a randomized crossover trial
Source: Front Endocrinol (Lausanne). 2023 Jan 9;13:1091421. doi: 10.3389/fendo.2022.1091421 (PMC9869378; doi:10.3389/fendo.2022.1091421)
Supplement: Supplementary file 1 [file Table_1.docx]

## Supplementary Table 1: Participant characteristics of those with positive screening diabetes antibodies

| Treatment Sequence | VP. | VP. | PV. | VP. | VP. | VP. | PV. | PV. | PV. |
| --- | --- | --- | --- | --- | --- | --- | --- | --- | --- |
| Ethnicity | NZE | NZE | Indian | M | P | P | M | NZE | M |
| C-peptide (pmol/L) | 523 | 320 | 821 | 1983 | 924 | 868 | 1096 | 1391 | 1268 |
| Combined antibody concentration (u/ml)  GAD IgG (IU/ml)  IA2 IgG (IU/mL)  ZnT8 (IU/mL) | 82  13  <10  <10 | 295  44  <10  <10 | 83  11  <10  <10 | 106  29  <10  <10 | 531  83  <10  <10 | 50  9  <10  <10 | 2293  406  <10  <10 | 20  6  <10  <10 | > Max  >2000  <10  <10 |
| Baseline HbA1c (mmol/mol [%]) | 82 [9.7] | 66 [8.2] | 75 [9.0] | 69 [8.5] | 70 [8.6] | 68 [8.4] | 63 [7.9] | 69 [8.5] | 79 [9.4] |
| Visit A HbA1c (mmol/mol [%]) | 69 [8.5] | 81 [9.6] | 61 [7.7] | 69 [8.5] | 77 [9.2] | 66 [8.2] | 78 [9.3] | 53 [7.0] | 62 [7.8] |
| Visit B HbA1c (mmol/mol [%]) | 82 [9.7] | 79 [9.4] | 56 [7.3] | WD | LTF | 65 [8.1] | 79 [9.4] | 58 [7.5] | 54 [7.1] |
| Change in HbA1c after pioglitazone (mmol/mol [%]) | 0 | +13 [1.2] | -19 [1.7] | - | - | -3 [0.3] | +15 [1.4] | -16 [1.5] | -17 [1.6] |
| Change in HbA1c after vildagliptin (mmol/mol [%]) | -13 [1.2] | +15 [1.4] | -14 [1.3] | 0 | +7 [0.6] | -2 [0.2] | +16 [1.5] | -11 [1.0] | -25 [2.2] |

V:vildagliptin; P: pioglitazone; NZE: New Zealand European; M: Māori, P: Pacific; WD: withdrawal; LTF: lost to follow up; GAD:glutamic acid decarboxylase; IA2: insulin autoantibody2; ZnT8: Zinc transporter
